# Supplementary material for: A new perleidid neopterygian fish from the Early Triassic (Dienerian, Induan) of South China, with a reassessment of the relationships of Perleidiformes
Source: PeerJ. 2022 May 17;10:e13448. doi: 10.7717/peerj.13448 (PMC9121871; doi:10.7717/peerj.13448)
Supplement: Supplemental Information 1 [file peerj-10-13448-s001.docx]

Online Supplementary Material

**A new perleidid fish from the Early Triassic of South China, with a reassessment of the relationships of Perleidiformes**

**Table of Contents**

1. Taxa and principal sources of data
2. Supplementary figure and table
3. Character list
4. Data matrix
5. References to supplementary information

**1. Taxa and principal sources of data**

FMNH, Field Museum of Natural History, Chicago, USA

IVPP, Institute of Vertebrate Paleontology and Paleoanthropology, Chinese Academy of Sciences, Beijing, China

LPV, Luoping County Vertebrates, Chengdu Institute of Geology and Mineral Resources, Chengdu, China

NHMUK, Natural History Museum, London, UK

PIMUZ, Paläontologisches Institut und Museum, Universität Zürich, Zürich, Switzerland

ZMNHM, Zhejiang Museum of Natural History, Hangzhou, China

*Moythomasia durgaringa*: Gardiner, 1984

*Australosomus kochi*: Nielsen, 1949; NHMUK P17141–17143, 17156, 17157, 17160, 17161, 20940–20945

*Acipenser brevirostrum*: Hilton et al., 2011

*Altisolepis bellipinnis*: Mutter, 2004; Sun et al., 2015

*Amia calva*: Grande and Bemis, 1998

*Boreosomus piveteaui*: Nielsen, 1942

*Brookvalia gracilis*: Hutchinson, 1973b

*Caturus furcatus:* FMNH UC2057; Patterson, 1975; Grande and Bemis, 1998

*Chondrosteus acipenseroides*: Hilton and Forey, 2009

*Cleithrolepidina minor*: Hutchinson, 1973b

*Cleithrolepis* *granulate*: Wade, 1935; Hutchinson, 1973b

*Colobodus baii*: Sun et al., 2008; IVPP V19974

*Colobodus bassanii*: Mutter, 2002, 2004

*Colobodus giganteus*: Cartanyà et al., 2015

*Chaohuperleidus* *primus* Sun et al., 2013; Dai et al., 2021

*Crenilepis sandbergeri*: Mutter, 2002, 2004

*Ctenognathichthys bellottii*: Bürgin, 1992

*Daedalichthys higginsi*: Hutchinson, 1973b

*Dipteronotus olgiatii*: Tintori, 1990

*Dipteronotus* *ornatus*: Bürgin, 1992

*Elops hawaiensis*: Forey, 1973; Sato & Yasuda, 1980

*Feroxichthys panzhouensis* Ma et al., 2021

*Feroxichthys yunnanensis* Xu, 2020

*Fuyuanperleidus dengi*: Geng et al., 2012; Sun et al., 2012

*Gabanellia* *agilis*: Tintori and Lombardo, 1996

*Gigantopterus teller*: Griffith, 1977

*Habroichthys broughi*: Lin et al., 2011; IVPP V19373–19380

*Helichthys browni*: Hutchinson, 1973b, 1978

*Helmolepis cyphognathus*: Neuman & Mutter, 2005

*Hydropessum* *kannemeyeri*: Hutchinson, 1973b

*Kyphosichthys grandei*: Xu & Wu, 2012

*Lepisosteus osseus*: Grande, 2010

*Leptolepis coryphaenoides*: Patterson, 1975; Arratia, 1999, 2013; Konwert & Stumpf, 2017

*Luganoia lepidosteoides*: Bürgin, 1992

*Luganoia* *fortuna*: Xu, 2020b

*Luopingichthys bergi*: Sun et al., 2009

*Louwoichthys pusillus*: Xu, 2021

*Meidiichthys browni*: Brough, 1931; Hutchinson, 1973b

*Moradebrichthys* *vilasecae* Cartanyà et al., 2019

*Paraperleidus* *changxingensis* Zhao and Lu, 2007; ZMNHM M1401

*Perleidus altolepis*: Lombardo, 2001

*Peipiaosteus pani*: Zhou, 1992

*Peltopleurus rugosus*: Bürgin, 1992; PIMUZ T2904

*Peltopleurus nitidus*: Xu and Zhao, 2016

*Peltopleurus tyrannos*: Xu et al., 2018

*Peltoperleidus* *ducanensis*: Bürgin et al., 1991

*Peltoperleidus macrodontus*: Bürgin, 1992

*Peltoperleidus obristi*: Herzog, 2001

*Peripeltopleurus hypsisomus*: Bürgin, 1992; PIMUZ T1211, 2150, 2869

*Pseudobeaconia elegans*: Hutchinson, 1973a; López-Arbarello and Zavattieri, 2008

*Platysiagum* *sinensis*: Wen et al., 2019

*Plesiofuro mingshuica*: Xu et al., 2015a

*Plesioperleidus* *yangtzensis* (Su, 1981) ZMNHM M8499, 8500; CUGM J2203a, K2-E2614; IVPP V5343;

*Polzbergia brochatus*: Griffith, 1977

*Potanichthys xingyiensis*: Xu et al., 2012

*Pteronisculus stensioi*: Nielsen, 1942; NHMUK P16282, 16283, 16300, 163001, 16307–16308

*Redifieldius gracilis*: Schaeffer and McDonald, 1978

*Semionotus elegans*: Olsen and McCune, 1991; Cavin, 2010; Grande, 2010; López-Arbarello, 2012

*Teffichthys* (*‘Perleidus’*) *madagascariensis*: NHMUK P16247, 16248, 19580–19584, 19587–19592, 19595–19599, 19603–19620, 19622, 19623; Lehman, 1952; Patterson, 1975; Marramà et al., 2017

*Thoracopterus niederristi*: Griffith, 1977; Lehman, 1979; NHMUK P1098

*‘Thoracopterus’ magnificus*: Tintori and Sassi, 1992

*‘Thoracopterus’ martinisi*: Tintori and Sassi, 1992

*Venusichthys comptus*: Xu and Zhao, 2016

*Wushaichthys exquisitus*: Xu et al., 2015b

**2. Supplementary Figure**

**
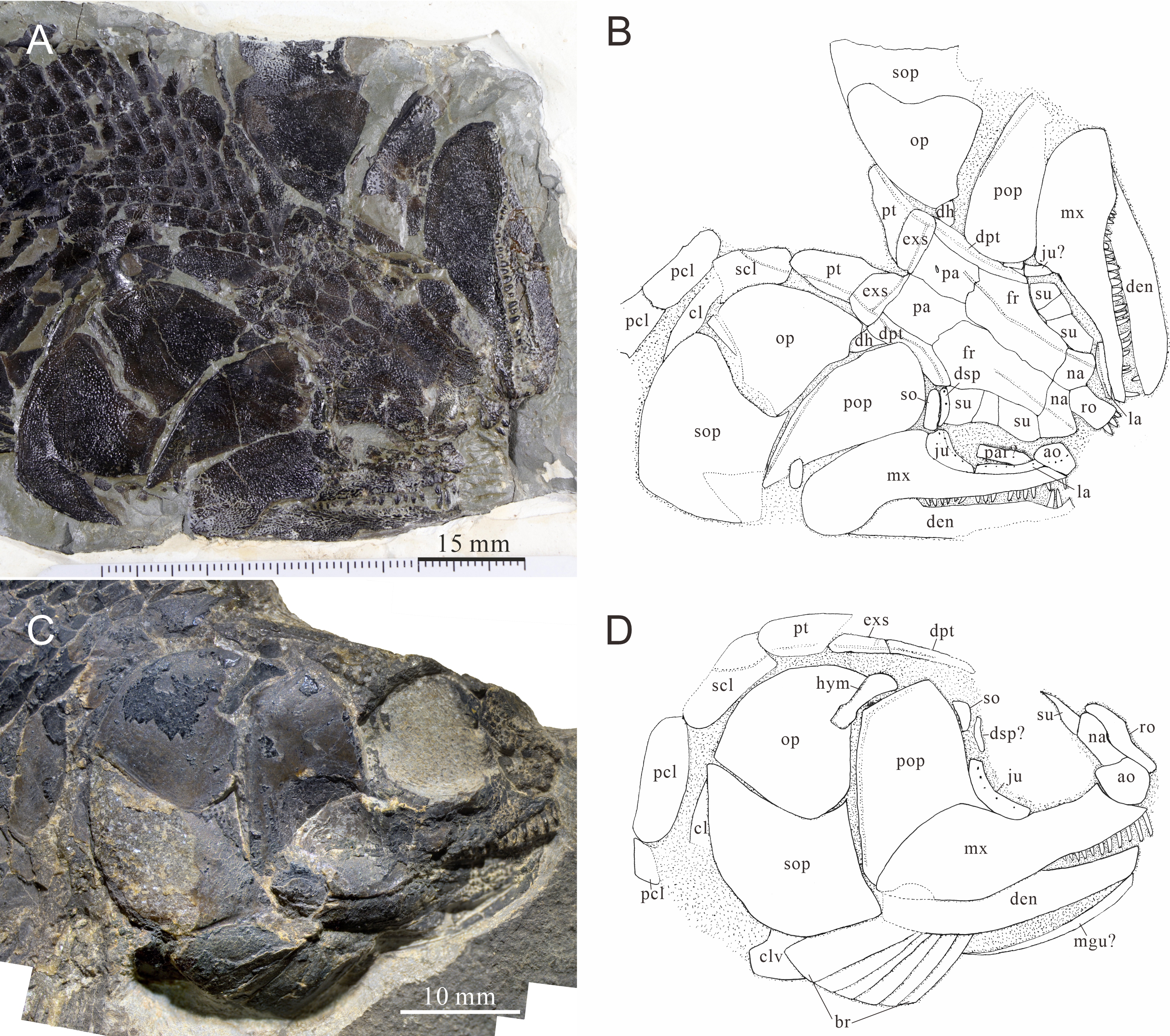
**

Supplementary Figure S1. Skull and pectoral girdle of *Paraperleidus* *changxingensis* and *Plesioperleidus yangtzensis*. A-B. *Paraperleidus* *changxingensis* Zhao and Lu, 2007, ZMNHM M1401; A. photo; B. ling-drawing. C-D: *Plesioperleidus* *yangtzensis* (Su, 1981), CUGM J2203a; C. photo; D. ling-drawing.

**
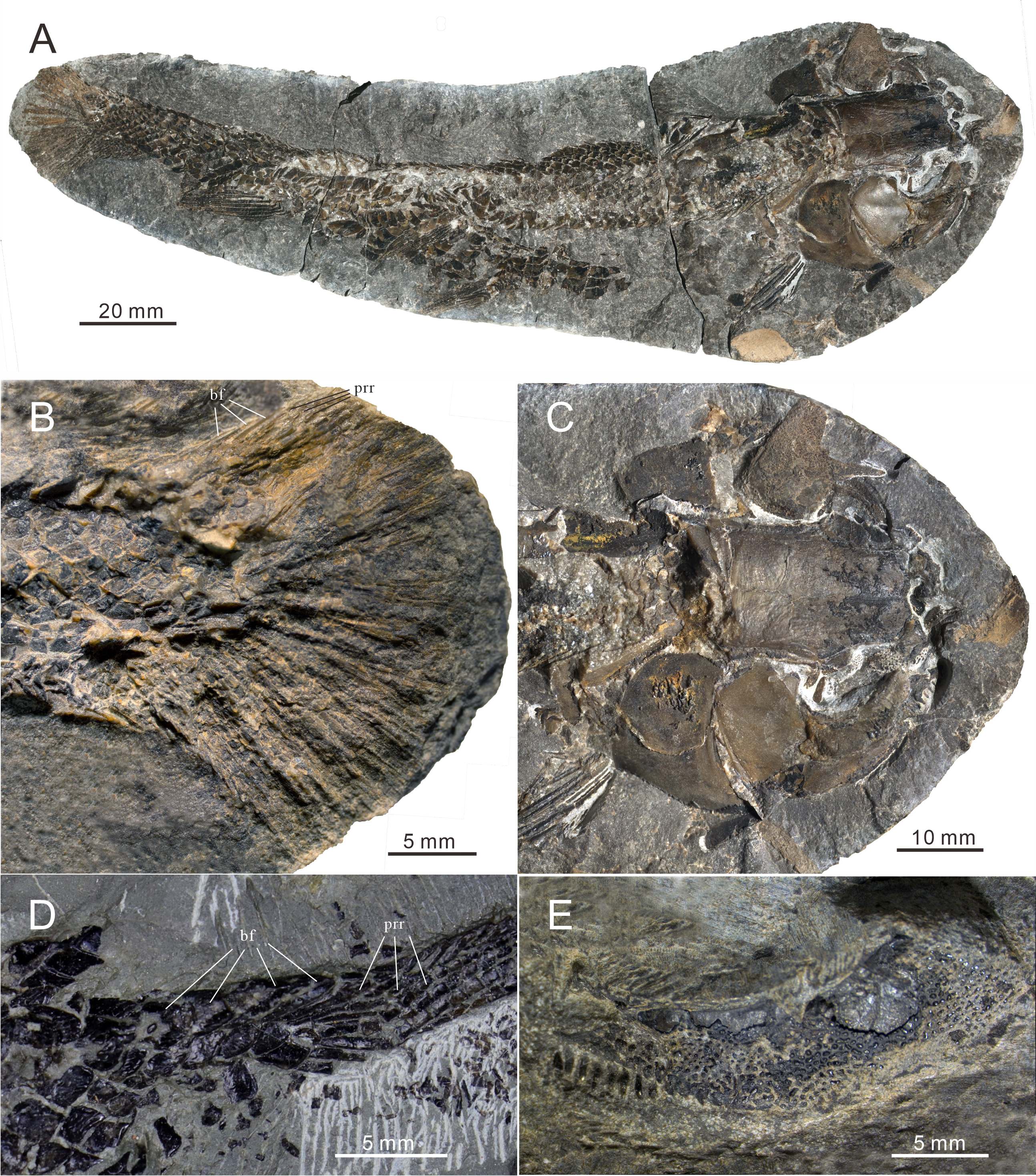
**

Supplementary Figure S2. A new specimen of *Plesioperleidus yangtzensis* and the caudal fin of *Paraperleidus changxingensis*. A-C. A new specimen of *Plesioperleidus* *yangtzensis* (CUGM K2-E2614). A. entire body; B. caudal fin; C. skull roof and cheek; D. the caudal fin of *Paraperleidus* *changxingensis*, ZMNHM M1401; E. the lacrimal fused with maxilla in *Plesioperleidus* *yangtzensis*, CUGM J2203a;
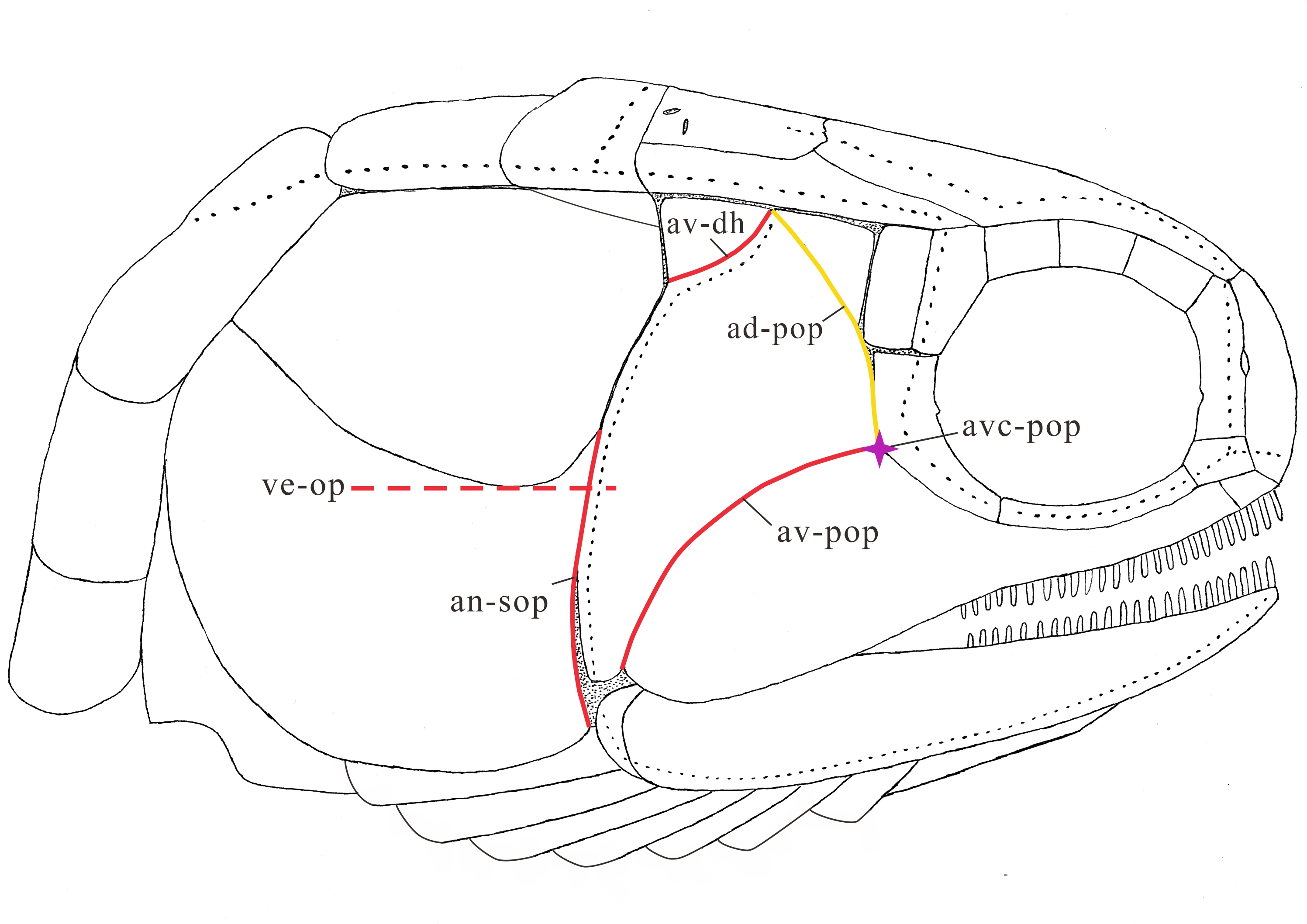


Supplementary Figure S3. The illustration of the added characters (take the *Teffichthys* *elegans* sp. nov. for example): ve-op: the ventral end of the opercle; ad-pop: the anterodorsal margin of the preopercle; av-pop: the anteroventral margin of the preopercle; avc-pop: anteroventral corner of the preopercle; av-dh: the anteroventral margin of the dermohyal; an-sop: the anterior margin of the subopercle.





Supplementary Figure S4. Strict consensus of 54 most parsimonious trees (tree length = 484 steps, consistency index = 0.401, retention index = 0.752), illustrating the phylogenetic position of *Teffichthys elegans* sp. nov. within the Neopterygii. Character changes indicated with solid circles are unique.

| Family | Put into Perleidiformes by | Put into other order or move out of Perleidiformes |
| --- | --- | --- |
| Teleopterinidae | Berg, 1940 | Amphicentriformes by Van, 2018 |
| Perleididae | Berg, 1940 |  |
| Cleithrolepidae | Berg, 1940; Hutchinson, 1973b | Polzbergiiformes by Xu, 2020b |
| Aetheodontidae | Gardiner, 1967 | *Aetheodontus* to Perleididae by Bürgin, 1992 |
| Hydropessidae | Hutchinson, 1973b | form a new clade *Hydropessum* (Cl*eithrolepis*+*Cleitrolepidina*) by López-Arbarello and Zavattieri, 2008 |
| Luganoiidae | Bürgin, 1992 | Luganoiiformes by Cartanya, 1999; Xu et al., 2015 |
| Platysiagidae | Bürgin, 1992 | Platysiagiformes by Griffith, 1977; Xu, 2020b |
| Gabanellidae | Tintori and Lomardo, 1996 |  |
| Polzbergiidae | Lombardo and Tintori, 2004 | Polzbergiiformes by Xu, 2020b |
| Colobodontidae | Mutter, 2004 | moved out by Xu, 2020a |
| Pseudobeaconiidae | López-Arbarello and Zavattieri, 2008 | Louwoichthyiformes by Xu, 2020b |
| Fuyuanperleididae | Sun et al., 2012 | Luganoiiformes by Xu, 2020b |
| Habroichthyidae | Tintori et al., 2016 | moved out by Xu, 2020b |

Supplementary Table T1. Summary of family variation in Perleidiformes.

**3.** **Character list**

**Skull Roof**

1. Rostral: large, shield-like or cap-like (0); small, much reduced or lost by fusion with other elements (1); irregularly shaped and anamestic (2). (Modified from Gardiner et al., 1996; Xu & Wu, 2012; Xu et al., 2015, 2018; Xu & Ma, 2016; Xu, 2020, 2021; Ma et al., 2021)
2. Postrostral: absent (0); present (1). (Xu, 2020, 2021; Ma et al., 2021)
3. Frontal(s): elongate (0); laterally expanded (1); well constricted above orbit (2). (Xu et al., 2012, 2015, 2018; Xu & Ma, 2016; Xu, 2020, 2021; Ma et al., 2021)
4. Contact relationships of frontals: anterior portions of frontals partly separated by median rostral bone (0); fully contact each other medially (1); paired, completely separated by rostral bones (2). (Xu, 2020; Ma et al., 2021)
5. Suture between frontals: present, paired frontals (0); absent, fusion of frontals into single element (1).
6. Supraorbital sensory canal: ending in parietal (0); ending in frontal (1); ending in dermopterotic (2). (Modified from Xu et al., 2012, 2015, 2018; Xu & Ma, 2016; Xu, 2020, 2021; Ma et al., 2021)
7. Distinct parietal: present (0); absent, fused with dermopterotic (1); absent, fused with frontal and dermopterotic (2); absent, fused with frontal (3). (Modified from Xu & Ma, 2016; Xu et al., 2018; Xu, 2020, 2021; Ma et al., 2021)
8. Suture between parietals: present (0); absent (1).
9. Number of parietals: one pair or single (0); three or more (1). (Modified from Xu, 2020, 2021; Ma et al., 2021)
10. Nasal bones: completely separated from each other by median rostral or rostral bones (0); joined or nearly joined in midline (1). (Xu & Ma, 2016; Xu et al., 2018; Xu, 2020, 2021; Ma et al., 2021)
11. Nasal bone forming part of orbital margin: present (0); absent (1). (Xu & Zhao, 2016; Xu & Ma, 2016; Xu et al., 2018; Xu, 2020, 2021; Ma et al., 2021)
12. Number of extrascapulars: two pairs or more (0); three (1); one pair (2). (Modified from Xu et al., 2012, 2015, 2018; Xu & Ma, 2016; Xu, 2020, 2021; Ma et al., 2021)
13. Intertemporal: present (0); absent (1). (Modified from Xu et al., 2015, 2018; Xu & Ma, 2016; Xu, 2020, 2021; Ma et al., 2021)
14. Ratio of dermopterotic length to parietal length: less than two (0); two or more (1). (Xu, 2020; Ma et al., 2021)
15. Accessory dermopterotic: absent (0); present (1). (Xu et al., 2015; Xu & Ma, 2016; Xu et al., 2018; Xu, 2020, 2021; Ma et al., 2021)
16. Dermopterotic/preopercle contact: present (0); absent (1). (Xu & Ma, 2016; Xu et al., 2018; Xu, 2020, 2021; Ma et al., 2021)

**Neurocranium**

1. Sphenotic with small dermal component: absent (0); present (1). (Grande, 2010; Xu & Wu, 2012; Xu & Ma, 2016; Xu et al., 2018; Xu, 2020, 2021; Ma et al., 2021)
2. Pterotic: present (0); absent (1). (Gardiner et al., 1996; Xu & Ma, 2016; Xu et al., 2018; Xu, 2020, 2021; Ma et al., 2021)
3. Opisthotic: present (0); absent (1). (Grande, 2010; Xu et al., 2015, 2018; Xu & Ma, 2016; Xu, 2020, 2021; Ma et al., 2021)
4. Intercalar: present (0); absent (1). (Gardiner et al., 1996; Xu & Ma, 2016; Xu et al., 2018; Xu, 2020, 2021; Ma et al., 2021)
5. Supraoccipital: absent (0); present (1). (Grande, 2010; Xu et al., 2015, 2018; Xu & Ma, 2016; Xu, 2020, 2021; Ma et al., 2021)
6. Post-temporal fossa: absent (0); present (1). (Gardiner & Schaeffer, 1989; Xu et al., 2015, 2018; Xu & Ma, 2016; Xu, 2020, 2021; Ma et al., 2021)
7. Sub-temporal fossa: absent (0); present (1). (Gardiner & Schaeffer, 1989; Xu et al., 2015, 2018; Xu & Ma, 2016; Xu, 2020, 2021; Ma et al., 2021)
8. Dilatator fossa: absent (0); present (1). (Gardiner & Schaeffer, 1989; Xu et al., 2015, 2018; Xu & Ma, 2016; Xu, 2020, 2021; Ma et al., 2021)
9. Posterior myodome: present (0); absent (1). (Modified from Gardiner & Schaeffer, 1989; Gardiner et al., 2005; Xu & Ma, 2016; Xu et al., 2018; Xu, 2020, 2021; Ma et al., 2021)
10. Anterodorsal myodome: present (0); absent (1). (Xu et al., 2014, 2018; Xu & Zhao, 2016; Xu & Ma, 2016; Xu, 2020, 2021; Ma et al., 2021)
11. Parasphenoid: short, terminates at otic fissure (0); long, extends across otic fissure (1). (Modified from Gardiner et al., 2005; Xu et al., 2015, 2018; Xu & Ma, 2016; Xu, 2020, 2021; Ma et al., 2021)
12. Basipterygoid process: present (0); absent (1). (Gardiner et al., 2005; Xu et al., 2015, 2018; Xu & Ma, 2016; Xu, 2020, 2021; Ma et al., 2021)
13. Internal carotid foramen on parasphenoid: absent (0); present (1). (Gardiner et al., 1996; Xu et al., 2015, 2018; Xu & Ma, 2016; Xu, 2020, 2021; Ma et al., 2021)
14. Efferent pseudobranchial foramen on parasphenoid: absent (0); present (1). (Gardiner et al., 1996; Xu et al., 2015, 2018; Xu & Ma, 2016; Xu, 2020, 2021; Ma et al., 2021)
15. Pineal foramen: present (0); absent (1). (Xu et al., 2014; Xu, 2020, 2021; Ma et al., 2021)

**Circumorbital Bones**

1. Anterior infraorbital bone(s): absent (0); present (1). (Modified from Grande, 2010; Xu & Wu, 2012; Xu et al., 2015, 2018; Xu & Ma, 2016; Xu, 2020, 2021; Ma et al., 2021)
2. Antorbital (when it is not fused with premaxilla): small, shorter than nasal (0); enlarged, nearly equal to or deeper than nasal (1). (Modified from Xu, 2020, 2021; Ma et al., 2021)
3. Tube-like canal bearing anterior arm of antorbital: absent (0); present (1). (Grande, 2010; Xu & Wu, 2012; Xu et al., 2015, 2018; Xu & Ma, 2016; Xu, 2020, 2021; Ma et al., 2021)
4. Anterior part of lacrimal bearing teeth: absent (0); present (1). (Modified from Xu, 2020, 2021; Ma et al., 2021)
5. Lacrimal: independent (0); fused with maxilla (1). (Xu, 2020; Ma et al., 2021)

Remarks: Based on personal observation on the holotype (IVPP V16517), the lacrimal is fused with the maxilla in *Fuyuanperleidus dengi* from the Middle Triassic (Anisian) of Luoping, Yunnan.

1. Dermosphenotic/nasal contact: present (0); absent (1). (Modified from Gardiner & Schaeffer, 1989; Xu et al., 2015, 2018; Xu & Ma, 2016; Xu, 2020, 2021; Ma et al., 2021)

Remarks: In *Helmolepis cyphognathus*, the triangular ‘supraorbital’ is better interpreted as a dermosphenotic according to its shape and position (Neuman & Mutter, 2005).

1. Dermosphenotic/preopercle contact: absent (0); present (1). (Xu, 2020, 2021; Ma et al., 2021)
2. Dermosphenotic attachment to skull roof in adult-sized individuals: loosely attached on the skull roof or hinged to the side of skull roof (0); firmly sutured into skull roof, forming part of it (1). (Grande & Bemis, 1998; Xu et al., 2015, 2018; Xu & Ma, 2016; Xu, 2020, 2021; Ma et al., 2021)
3. Position of dermosphenotic relative to dermopterotic (intertemporal plus supratemporal): located at nearly same horizontal level of dermopterotic (0); located below dermopterotic (1). (Ma et al., 2021)
4. Suborbital(s): absent (0); present (1). (Modified from Gardiner & Schaeffer, 1989; Xu & Ma, 2016; Xu et al., 2018; Xu, 2020, 2021; Ma et al., 2021)

Remarks: The coding (1) for *Australosomus* is based on personal observation on NHMUK 17157.

1. Positions of suborbital(s): extending below dermosphenotic (0); located posterior to dermosphenotic only (1). (Modified from Xu, 2020, 2021; Ma et al., 2021)
2. Number of infraorbitals between antorbital and dermosphenotic: three or less (0); four or more (1). (Modified from Gardiner & Schaeffer, 1989; Xu et al., 2015, 2018; Xu & Ma, 2016; Xu, 2020, 2021; Ma et al., 2021)
3. Postinfraorbital(s): absent (0); present (1). (Xu, 2020, 2021; Ma et al., 2021)
4. Supraorbital: absent (0); present (1). (Modified from Gardiner & Schaeffer, 1989; Xu et al., 2015, 2018; Xu & Ma, 2016; Xu, 2020, 2021; Ma et al., 2021)
5. Number of supraorbital bones: single (0); two (1); three or more (2). (Modified from Gardiner & Schaeffer, 1989; Xu et al., 2015, 2018; Xu & Ma, 2016; Xu, 2020, 2021; Ma et al., 2021)

Remarks: Based on personal observation (IVPP V25666), four supraorbital bones are present in *Luopingichthys bergi* from the Middle Triassic (Anisian) of Luoping, Yunnan.

1. Multiple supraorbital bones arranged in more than one horizontal rows: absent (0); present (1). (Xu, 2020; Ma et al., 2021)

**Jaws and dentation**

1. Premaxilla: fused with antorbital, bearing sensory canal (0); present as distinct elements, lacking sensory canal (1); lost (2); fused with rostral, bearing sensory canal (3). (Modified from Xu & Ma, 2016; Xu et al., 2018; Xu, 2020, 2021; Ma et al., 2021)

Remarks: Personal reexaminations on the type specimens (LPV-11014 and LPV-11797) confirm that a pair of small, toothed premaxillae lacking sensory canal is present in *Platysiagum sinensis*.

1. Premaxilla(e): present as a pair of elements (0); fused as a median element (1). (Modified from Xu & Ma, 2016; Xu et al., 2018; Xu, 2020, 2021; Ma et al., 2021)
2. Mobile premaxilla: absent (0); present (1). (Modified from Gardiner & Schaeffer, 1989; Xu & Ma, 2016; Xu et al., 2018; Xu, 2020, 2021; Ma et al., 2021)
3. Premaxilla immovably attached to braincase by means of a deep nasal process tightly sutured to frontals: absent (0); present (1). (Grande, 2010; Xu & Wu, 2012; Xu et al., 2015, 2018; Xu & Ma, 2016; Xu, 2020, 2021; Ma et al., 2021)
4. Number of marginal teeth on premaxilla: three or more (0); one or two (1). (Xu, 2020, 2021; Ma et al., 2021)
5. Foramen for olfactory nerve on premaxilla: absent (0); present (1). (Modified from Grande, 2010; Xu & Wu, 2012; Xu et al., 2015, 2018; Xu & Ma, 2016; Xu, 2020, 2021; Ma et al., 2021)
6. Maxilla/preopercle contact: present (0); absent (1). (Coates, 1999; Xu & Ma, 2016; Xu et al., 2018; Xu, 2020, 2021; Ma et al., 2021)
7. Ventral portion of preopercle anteriorly extended, contacting maxilla anteriorly: absent (0); present (1). (Modified from Xu, 2020, 2021; Ma et al., 2021)
8. Supramaxilla: absent (0); present (1). (Modified from Gardiner & Schaeffer, 1989; Xu & Ma, 2016; Xu et al., 2018; Xu, 2020, 2021; Ma et al., 2021)
9. Number of supramaxilla: single (0); two (1). (Xu & Zhao, 2016; Xu & Ma, 2016; Xu et al., 2018; Xu, 2020, 2021; Ma et al., 2021)
10. Maxilla: present (0); absent (1). (Xu & Ma, 2016; Xu et al., 2018; Xu, 2020, 2021; Ma et al., 2021)
11. Mobile maxilla in cheek: absent (0); present (1). (Coates, 1999; Xu & Ma, 2016; Xu et al., 2018; Xu, 2020, 2021; Ma et al., 2021)
12. Suborbital/maxilla contact: present (0); absent (1). (Modified from Cloutier & Arratia, 2004; Xu et al., 2015, 2018; Xu & Ma, 2016; Xu, 2020, 2021; Ma et al., 2021)
13. Expanded dorsal lamina of maxilla: present (0); absent (1). (Modified from Cloutier & Arratia, 2004; Xu, 2020, 2021; Ma et al., 2021)
14. Depth of dorsal lamina of maxilla: no smaller than orbital length (0); smaller than orbital length (1). (Xu, 2020, 2021; Ma et al., 2021)
15. Posterior margin of maxilla: slightly convex or straight (0); concave with a posterior maxillary notch (1). (Grande & Bemis, 1998; Xu & Wu, 2012; Xu et al., 2015, 2018; Xu & Ma, 2016; Xu, 2020, 2021; Ma et al., 2021)
16. Oral margin of maxilla: concave or nearly straight (0); convex (1). (Xu, 2020, 2021; Ma et al., 2021)
17. Posterior end of maxilla relative to orbit: well behind orbit (0); ending below (or nearly below) posterior orbital margin (1); ending nearly below orbital center or even anteriorly located (2). (Xu, 2020, 2021; Ma et al., 2021)
18. Teeth on maxilla: present (0); much reduced or lost (1). (Modified from López-Arbarello & Zavattieri, 2008; Xu & Ma, 2016; Xu et al., 2018; Xu, 2020, 2021; Ma et al., 2021)
19. Distribution of teeth on maxilla: most of oral margin of maxilla (0); only anterior portion of oral margin of maxilla (1). (Xu, 2020, 2021; Ma et al., 2021)
20. Extraordinarily long, fang-like teeth on both jaws: absent (0); present (1). (Xu, 2020, 2021; Ma et al., 2021)
21. Molariform teeth on coronoid(s), prearticular and palatine bones: absent (0); present (1). (Xu, 2020; Ma et al., 2021)
22. Teeth on dentary: present (0); absent (1). (Xu & Ma, 2016; Xu et al., 2018; Xu, 2020, 2021; Ma et al., 2021)
23. Quadratomandibular articulation: well behind orbit or below posterior orbit margin (0); nearly below the orbital center or even anteriorly located (1). (Modified from López-Arbarello & Zavattieri, 20082; Xu et al., 2012, 2015, 2018; Xu & Ma, 2016; Xu, 2020, 2021; Ma et al., 2021)
24. Coronoid process: absent (0); present (1). (Gardiner & Schaeffer, 1989; Xu et al., 2015, 2018; Xu & Ma, 2016; Xu, 2020, 2021; Ma et al., 2021)
25. Supra-angular bone in lower jaw: present (0); absent (1). (Arratia, 2013; Xu & Ma, 2016; Xu et al., 2018; Xu, 2020, 2021; Ma et al., 2021)

**Palatoquadrate, Hyoid Arch, and Operculo-gular Series**

1. Vomers in adults: paired (0); fused (1). (Xu & Ma, 2016; Xu et al., 2018; Xu, 2020, 2021; Ma et al., 2021)
2. Suspensorium angle: acute (0); nearly vertical (1). (Modified from Gardiner et al., 2005; Xu et al., 2015; Xu & Ma, 2016; Xu et al., 2018; Xu, 2020, 2021; Ma et al., 2021)
3. Hyomandibula with canal for hyoid branch of nerve VII: absent (0); present (1). (Modified from Coates, 1999; Xu et al., 2015; Xu & Ma, 2016; Xu et al., 2018; Xu, 2020, 2021; Ma et al., 2021)
4. Dermohyal: present (0); absent (1). (Modified from Gardiner et al., 2005; Xu & Ma, 2016; Xu et al., 2018; Xu, 2020, 2021; Ma et al., 2021)
5. Postspiracle: absent (0); present (1). (Xu & Ma, 2016; Xu et al., 2018; Xu, 2020, 2021; Ma et al., 2021)
6. Quadratojugal: plate-like (0); splint-like (1); much reduced or lost (2). (Modified from Gardiner et al., 2005; Coates, 1999; Xu et al., 2015; Xu & Ma, 2016; Xu et al., 2018; Xu, 2020, 2021; Ma et al., 2021)
7. Symplectic: absent (0); present (1). (Modified Gardiner et al., 2005; Xu et al., 2015; Xu & Ma, 2016; Xu et al., 2018; Xu, 2020, 2021; Ma et al., 2021)
8. Symplectic involvement of jaw joint: absent (0); present (1). (Grande & Bemis, 1998; Grande, 2010; Xu & Wu, 2012; Xu et al., 2015, 2018; Xu & Ma, 2016; Xu, 2020, 2021; Ma et al., 2021)
9. Elongated posteroventral process of quadrate: absent (0); present (1). (Modified from Gardiner et al., 1996; Xu et al., 2015, 2018; Xu & Ma, 2016; Xu, 2020, 2021; Ma et al., 2021)
10. Number of hypobranchials: three (0); four (1). (Grande, 2010; Xu & Wu, 2012; Xu et al., 2015, 2018; Xu & Ma, 2016; Xu, 2020, 2021; Ma et al., 2021)
11. Uncinate processes on epibranchials: absent (0); present (1). (Coates, 1999; Xu et al., 2015, 2018; Xu & Ma, 2016; Xu, 2020, 2021; Ma et al., 2021)
12. Interopercle: absent (0); present (1). (Gardiner & Schaeffer, 1989; Xu et al., 2015, 2018; Xu & Ma, 2016; Xu, 2020, 2021; Ma et al., 2021)
13. Two or more preopercular elements on each side of skull: absent (0), present (1). (Xu & Zhao, 2016; Xu & Ma, 2016; Xu et al., 2018; Xu, 2020, 2021; Ma et al., 2021)
14. Preopercle: present (0); absent (1). (Xu, 2020, 2021; Ma et al., 2021)
15. Shape of preopercle: boomerang-shaped or irregular (0); crescent-shaped or L-shaped (1). (Modified from Xu & Zhao, 2016; Xu & Ma, 2016; Xu et al., 2018; Xu, 2020, 2021; Ma et al., 2021)
16. Ventral end of preopercle located well above posterior end of oral margin of maxilla (when the ventral part of preopercle contacts maxilla): present (0); absent (1). (Modified from Xu, 2020, 2021)
17. Opercle: present (0); absent (1). (Xu, 2020, 2021; Ma et al., 2021)
18. Size of opercle: significantly larger than subopercle (0); nearly equal to or smaller than subopercle (1); much reduced (2). (Modified from Xu & Wu, 2012; Xu et al., 2015, 2018; Xu & Ma, 2016; Xu, 2020, 2021; Ma et al., 2021)
19. Suture between opercle and subopercle: slightly inclined or horizontal (0); greatly inclined (1). (Xu & Ma, 2016; Xu et al., 2018; Xu, 2020, 2021; Ma et al., 2021)
20. Prominent anterodorsal process of subopercle: absent (0); present (1). (Xu, 2020; Ma et al., 2021)
21. Prominent anteroventral extension of subopercle: absent (0); present (1). (Xu, 2020, 2021; Ma et al., 2021)
22. Number of branchiostegal rays: ten or more (0); seven to nine (1); four to six (2); three (3); two (4); single (5). (Modified from Xu, 2020, 2021; Ma et al., 2021)
23. Posterior-most branchiostegal ray: elongate, plate-like or rod-like (0); triangular, much expanded posteriorly (1).
24. Gular bone(s): present (0); absent (1). (Modified from Coates, 1999; Xu et al., 2015, 2018; Xu & Ma, 2016; Xu, 2020, 2021; Ma et al., 2021)

**Vertebrate and Caudal Skeleton**

1. Solid vertebral centra of adult-sized individuals: absent (0); present (1). (Modified from Grande & Bemis, 1998; Xu et al., 2015, 2018; Xu & Ma, 2016; Xu, 2020, 2021; Ma et al., 2021)
2. Epipleural intermuscular bones: absent (0); present (1). (Arratia, 2013; Xu & Ma, 2016; Xu et al., 2018; Xu, 2020, 2021; Ma et al., 2021)
3. Uroneural: absent (0); present (1). (Xu & Wu, 2012; Xu & Ma, 2016; Xu et al., 2018; Xu, 2020, 2021)
4. Division of hypurals into dorsal and ventral groups (a gap between hypurals 2 and 3): absent (0); present (1). (Xu & Ma, 2016; Xu et al., 2018; Xu, 2020, 2021; Ma et al., 2021)

**Girdles and Fins**

1. Posttemporal: contacting extrascapular posteriorly (0); contacting extrascapular posterolaterally and separating this bone from contact with its counterpart (1); contacting extrascapular medially, and being incorporated into the skull roof (2); lost (3). (Modified from Xu et al., 2012, 2015, 2018; Xu & Ma, 2016; Xu, 2020, 2021; Ma et al., 2021)
2. Width of posttemporals: broad, nearly as wide as extrascapular (0); relatively narrow, about half width of extrascapular series (1). (Xu, 2020, 2021; Ma et al., 2021)
3. Clavicle: present as a broad plate (0); much reduced or lost (1). (Modified from Gardiner et al., 1996; Xu et al., 2015, 2018; Xu & Ma, 2016; Xu, 2020, 2021; Ma et al., 2021)
4. Supracleithrum relative to posterior margin of opercle in depth: supracleithrum nearly as deep as posterior margin of opercle (0); supracleithrum shorter than posterior margin of opercle (1); supracleithrum well deeper than posterior margin of opercle (2). (Ma et al., 2021)
5. Pectoral fins enlarged as wings: absent (0); present (1). (Xu et al., 2012, 2018; Xu & Ma, 2016; Xu, 2020, 2021; Ma et al., 2021)
6. Pelvic fins enlarged as auxiliary wings: absent (0); present (1). (Xu et al., 2012, 2018; Xu & Ma, 2016; Xu, 2020, 2021; Ma et al., 2021)
7. Number of dorsal and anal fin rays relative to radials: rays more numerous than radials (0); rays and radials nearly equal in number (1). (Gardiner et al., 2005; Xu et al., 2015, 2018; Xu & Ma, 2016; Xu, 2020, 2021; Ma et al., 2021)

Remarks: In *Cleithrolepis granulate*, the rays are more numerous than the radials in both the dorsal (19 radials corresponding to 27 rays) and anal (9 radials corresponding to 19 rays) fins (Wade, 1935). The similar condition is also present in *Polzbergia brochatus*, in which, the rays are also more numerous than the radials in the dorsal fin (Griffith, 1977).

1. Dorsal and anal fin rays: segmented throughout the length (0); segmented distally (1). (Xu & Gao, 2011; Xu et al., 2015, 2018; Xu & Ma, 2016; Xu, 2020, 2021; Ma et al., 2021)
2. Origin of dorsal fin: nearly opposite to the origin of anal fin, well posterior to the origins of pelvic fins (0); between origins of anal and pelvic fins (1); anterior to origins of pelvic fins (2). (Xu, 2020, 2021; Ma et al., 2021)
3. Caudal fin rays: terminate at caudal extremity of body axis (0); extend beyond termination of body axis (1). (Modified from Gardiner et al., 2005; Xu et al., 2015, 2018; Xu & Ma, 2016; Xu, 2020, 2021; Ma et al., 2021)
4. Epaxial procurrent rays in caudal fin: absent (0); present (1). (Modified from López-Arbarello & Zavattieri, 2008; Xu, 2020, 2021; Ma et al., 2021)
5. Number of epaxial procurrent rays in caudal fin: four or more (0); one to three (1). (Xu, 2020; Ma et al., 2021)
6. Number of principal caudal fin rays: 25 or more (0); 24 or less (1). (Xu, 2020, 2021; Ma et al., 2021)
7. Principal rays of caudal fin ornamented with rounded ganoid tubercles: absent (0); present (1). (Xu, 2020)
8. Dense lepidotrichial segments of pectoral fin rays between innermost principal pectoral fin ray and body: absent (0); present (1). (Xu et al., 2012, 2015, 2018; Xu & Ma, 2016; Xu, 2020, 2021; Ma et al., 2021)
9. Dense brush-like rays proximally articulating several stout segments at posterior portion of male anal fin: absent (0); present (1). (Modified from Xu & Ma, 2016; Xu et al., 2018; Xu, 2020, 2021; Ma et al., 2021)
10. Modification of male anal fin into unsegmented rays with tiny hooklets along anterior margin of longest leading ray: absent (0); present (1). (Xu & Ma, 2016; Xu et al., 2018; Xu, 2020, 2021; Ma et al., 2021)
11. Enlarged and posteriorly extended lateral scutes associate with anal fin: absent (0); present (1). (Modified from Xu & Ma, 2016; Xu et al., 2018; Xu, 2020, 2021; Ma et al., 2021)
12. Anal fin relative to dorsal fin in size: anal fin smaller than or equal to dorsal fin (0); anal fin larger than dorsal fin (1). (Xu & Ma, 2016; Xu et al., 2018; Xu, 2020, 2021; Ma et al., 2021)
13. Caudal fin: forked, lower lobe slightly shorter than or largely equal to upper lobe (0); forked, lower lobe longer than upper lobe (1); unforked (2). (Modified from Xu et al., 2012, 2015, 2018; Xu & Ma, 2016; Xu, 2020, 2021; Ma et al., 2021)
14. Number of epaxial basal fulcra associated with caudal fin: six or more (0); five or less (1).
15. Fringing fulcra on caudal fin: present (0); absent (1). (Xu & Ma, 2016; Xu et al., 2018; Xu, 2020, 2021; Ma et al., 2021)
16. Fringing fulcra on pectoral fins: present (0); absent (1). (Xu & Ma, 2016; Xu et al., 2018; Xu, 2020, 2021; Ma et al., 2021)

**Body shape and scales**

1. An apparent dorsal hump between head and dorsal fin: absent (0); present (1). (Xu & Wu, 2012; Xu, 2020, 2021; Ma et al., 2021)
2. Scales: present (0); absent (1). (Modified from Xu & Ma, 2016; Xu et al., 2018; Xu, 2020, 2021; Ma et al., 2021)
3. Anterior flank scales: present (0); much reduced or absent (1). (Modified from Xu & Ma, 2016; Xu et al., 2018; Xu, 2020, 2021; Ma et al., 2021)
4. Lateral line scales: the lateral line scales as deep as or slightly deeper than those scales above and below (0); greatly deepened, 30% or more of the greatest depth of the body (1). (Modified from Xu & Ma, 2016; Xu et al., 2018; Xu, 2020, 2021; Ma et al., 2021)
5. Two horizontal rows of scales notably deepened in anterior flank region (lateral line scales notably deepened and nearly equal to the scales ventral to them): absent (0); present (1). (Xu, 2020; Ma et al., 2021)
6. Greatly deepened anterior flank scales corresponding to two or three horizontal rows of relatively shorter scales posteriorly: absent (0); present (1). (Xu, 2020; Ma et al., 2021)
7. Rhomboidal scales in anterior flank region: ornamented with ganoid ridges and tubercles (0); nearly smooth (1). (Xu, 2020; Ma et al., 2021)
8. Type of scales: ganoid of lepisosteoid type (0); elasmoid of amioid type (1); elasmoid of cycloid type (2). (Xu et al., 2012, 2015, 2018; Arratia, 2013; Xu & Ma, 2016; Xu, 2020, 2021; Ma et al., 2021)
9. A posteriorly directed spine on dorsal ridge scale anterior to dorsal fin: absent (0); present (1). (GML36; López-Arbarello & Zavattieri, 2008; Xu & Wu, 2012; Xu, 2020, 2021; Ma et al., 2021)
10. Posteriorly inclined scales in pectoral region: absent (0); present (1). (López-Arbarello & Zavattieri, 2008; Xu & Ma, 2016; Xu et al., 2018; Xu, 2020, 2021; Ma et al., 2021)

(135) The length of the anteroventral margin of dermohyal/the length of the anterodorsal margin of preopercle: more than half (0); nearly half (1); less than half (2).

(136) The position of anteroventral corner of preopercle compared with the ventral end of opercle: upper (0); nearly at the same level (1); lower (2)

(137) The suborbital nest the posterior margin of dermosphenotic: nearly triangular (0); slender, nearly the same depth with each other (1); broad, nearly the same depth with each other (2); enlarged, nearly half the depth of preopercle (3); formed small series (4).

(138) Number of suborbitals: single (0); two (1); three or more (2). The spiracular in *Teffichthys* is in the same position with suborbital in other taxa, like *Peltoperleidus*. Accordingly, the coding for *Teffichthys* is '1'.

(139) Epaxial principal rays in caudal fin: absent (0); present (1).

(140) The caudal fin type: true heterocercal (0); abbreviated heterocercal (1); homocercal (2); asymmetrical, lower lobe longer than upper lobe (3).

(141) The length of the anteroventral margin length of preopercle compared with the length of the anterior margin of subopercle: the anterior is longer than the latter (0), nearly the same (1), the anterior is shorter than the latter (2). When the preopercle is not contact with the maxilla, the coding for the character is '-'.

(142) The length of the anteroventral margin of preopercle /the length of the anterodorsal margin of preopercle: >2 (0), 1-2 (1), ~1 (2), 0.5-1 (3), <0.5 (4). When the preopercle is not contact with the maxilla, the coding for the character is '-'.

**4. Data matrix**

*Moythomasia*
0000000000000-0-0----00000000000-00000000-000--000000000-00-0000000000000001000000000000000000000000000000000000-00000000000000000000001--0000

*Pteronisculus*
0000000000000-0-0----000000000100010000010010--2-00-0000-0000100000000000001000000000000000000000000000000000100-00000000000000000000000310000

*Boreosomus*_*piveteaui*
0000000000000-0-0----000000010100000100010010--2-00-0000-001010000000000000000?000000000020000000000000010000200-00000000000000000000001-20003

*Acipenser*
2102000000111-0-0001000010110010?0?-10000---1002-00----0-1----------0-001-0010000000001--1--003010000200-0000100-00000000001000----000----00--

*Chondrosteus*_*acipenseroides*
2?01000000001-0-0???0?????1?0?1????-10000---0--2-00----0-1----------0-001-00100000??001--02000101000001000000200-00000000001001----000----00--

*Peipiaosteus*_*pani*
2?010?00001?1-0-0???0?????1????????-10000---0--2-00----0-1----------0-001-00100000??001--0200020100002?000000100-00000000001001----000----00--

*Australosomus*
00000000101210000???001101010010??00?0-010010--???0?0000-0010100000000000001000000010000000000100000000010000011000000010011000200100010??1201

*Brookvalia*_*gracilis*
01000200101210000???0?????????101000100010011002-00--000-000010000000000??0?002?????000000100050?????00020000010-00000000000000000000001?00100

*Daedalichthys*_*higginsi*
01000100001210000???0?????????101000100010011002-00--000-00?010000000000??0?002?????000000100040?????00020000010-00000000000000000000001?00100

*Redfieldius*_*gracilis*
0100010-101010010???0?????????101000100010011002-00--000-001010000000000??0?002?????000000100050?????00020000011000000000000000000100001011100

*Helichthys*_*browni*
01000100101210010???0?????????101000100010011002-00--000-001010000000000??0?002?????000000100050?????00020000011000000000000000000100001001100

*Platysiagum*
00010000010210000???0?????????10000011000-000--10?000000-001010000000000??1?00??????00000011001000???00000000110-00000000000000000(01)00011--0113

*Helmolepis*_*cyphognathus*
00010000010010000???0?????????10000001000-000--??????000-001010000000000??1?00??????00000001001000???00000000110-00000000000000000100021--0113

*Cleithrolepidina*
00010000000211100???0?????????100000110111?0120??00-0000-001010011000100??1?000?????0000001000???0???3-?100?011??0000000010010000000012200?124

*Cleithrolepis*
000?0000000011100???0?????????100000110111001201000-0000-001010011000(01)00??1?000?????000000100000?0???3-??000011100000000010?100000000122001124

*Polzbergia*
?00?00000??21?000???0?????????1???001101????1201000000?0-001010?1100000???1?00??????0?00?0000????????3-01000001100000000010?000000000022??1124

*Hydropessum*
00010?00000211000?????????????100?00110111001101000-?000-001010011-0?1??????10??????0?0000?000???????00?000??111?10000000?0?10000000001-001124

*Colobodus*_*giganteus*
01000000000211000???0?????????1000??11010-00121100000000-00-010000001000??1?002?????000000001000?????0??000?111110?000000?000000000000?0--1102

*Colobodus*_*bassanii*
01000000000211000???0?????????10000011010-001201000?0000-00-0100000010000?11002?????00000000100000???01?000111111010000001000000000000?0--1102

*Colobodus*_*baii*
01000000000211000???0?????????1000001?0?0-001?1100000000-00-0100000010000?11002?????00000000?0?0?????01?000?1111001000000100000000000020--1101

*Crenilepis*_*sandbergeri*
01000000000211000???0?????????10000011010-00121100000000-00-0100000010000?11002?????00000000100000???01?000111110010000001000000000000?0--?102

*Feroxichthys*_*yunnanensis*
0000001000021-000???0?????????1000-111010-00121110000000-00-010000101000??1?002?????0000000010100????01?000?11110?1000000000000000100020--?122

*Feroxichthys*_*panzhouensis*
??????????????????????????????????-111010-00121??????000-00-0001001010000?1?002?????0000000010100???????000?11110010000?0000100000000020--1122

*Chaohuperleidus*
000?001000021-000?????????????100?001001100111010000?010-00101000010?0?0????00??????0000000010000????01?00011111001000000000000000100021??1122

*Gabanellia*
00000?00000211000?????????????100?00100011111201000??0?0-00101000010?0?0????10??????0000000010000????01??00?111100?0101001000000000000-0001202

*Plesiofuro*
00010000000211000???0?????????10000010011100120100000000-0010100000000000?1?002?????00000010002000???00?00011110-10000000000000000100011100111

*Meidiichthys*_*browni*
00010000000211000???0?????????10000010011100120100000000-00101000000?000??1?002??00?0000001000200????00000011110-100000000000000001000111001??

*Pleisioperleidus*_*yangtzensis*
0001001000021-00???????????????00?0(01)10011100120?0000?010-00101000000?0?0????00??????0000001000200????00?00011111???000000?000000001000111?0112

*Paraperleidus*_*changxingensis*
0001000001021100???????????????00?0010011100120100000000-00101000000?000????00??????0000001000???????00?000????11??0??????0000000010001?100111

*Teffichthys*_*elegans*
0001000000021100???????????????00?0010011100120100000000-0010100000010?0????01??????0000001000200????01?00011111110000000000000000100011110111

*Teffichthys*_*madagascariensis*
00010000000211000---01110?100010000010011100120100000000-001010000001000??11002??00?00000010002000???01?00011111110000000000000000100011110111

*Perleidus*_*altolepis*
00010000000211000???0?????????10000010011100120100000000-001010000001000??1?002?????0000001000200????01?00011111010000000000000000100011101111

*Peltoperleidus*_*ducanensis*
000111010?0210000???0?????????100000100111001?0100000010-00101001010?000??1?002?????0000001001410????01?00011110-10000000000000010100022110124

*Peltoperleidus*_*macrodontus*
00011011-00?1-000???0?????????10000010011100120100010010-00101001010100???1?002?????000000100141?????01?00011110-10000000000000010100022110124

*Peltoperleidus*_*obristi*
00011101000210000???0?????????10000010011100120100000010-00101001010?000??1?002?????0000001001410????01?000?1110-100000000000000101000?2??0124

*Peltoperleidus*_*asiaticus*
00011-(23)1-0021-000???0?????????10000010011100120100010010-001010010101000??1?002?????0000001001410????010000?1110-10000000000000000100022110124

*Pseudobeaconia*_*elegans*
01010000000010000???0?????????10000011010-00110100000010-001010010101000??1?002?????0000001001300????0100001111111000000010?000000101022--1124

*Dipteronotus*_*olgiatii*
00010000000211000???0?????????10000010011100100??????010-00101001010?000??1???2?????0000?01001???????0??000?111101000000010?1000001010?2001123

*Dipteronotus*_*ornatus*
0001000000?211000???0?????????1000001001??001101010?0010-001010010101000??1???2?????0000?0100130????????000?1111??0000000?0?1000000010?2??1123

*Louwoichthys*_*illus*
0001(01)0(02)(01)000210000???0?????????10000010011100120100010010-0010100101100000?1?002?????00000010014100???01000011111010000000100000000100022001124

*Ctenognathichthys*
00010000000210000???0?????????10000010011100120100010010-001000010110001??1?002?????0000001001(34)1?????01?00011111000000000100000000100022??1124

*Luopingichthys*_*bergi*
00010000000210000???0?????????10000010011100120100010010-0010100101100010?1?002?????00000010014100???01000011111000000000100000000100022??1124

*Luganoia*_*lepidosteoides*
1021001000001-010???0?????????1000-110011000121310000000-000010020101010??11002?????0100-01001?0?????01?10011211010000002111000101100022121113

*Luganoia*_*fortuna*
1021001000011-010???0?????????1000-1100110001??310000000-00001002010?010??1?002?????0000-01001???????01?10011211010000002111000101100022221112

*Fuyuanperleidus*_*dengi*
0001001000021-000???0?????????100001100111001201000?0000-0010100001010000?1?002?????0000000000200????01?100111110000000001110001011000?2001113

*Altisolepis*_*bellipinnis*
00011000000210000???0?????????10000010011100120??????000-001010000000000??11?02?????0000000000200????01?100?11110100000101110001001000?2??1224

*Peltopleurus*_*nitidus*
0001(01)1(02)(01)000110010???0?????????10000010011100120100000000-00101000010?000??1?012?????0000100000300????01010011111010010110100000100100021200112

*Peltopleurus*_*tyrannos*
0001(01)1(02)(01)000010010???0?????????10000010011100110100000000-001010000101000??1?012?????0000100000200????0??100?1111010010110100000100100020200112

*Peltopleurus*_*rugosus*
00010100000110010???0?????????10000010011100110??00?0000-00101000010?000??1?0?2?????0000100000200????01?100111110100101001010001001000-1421102

*Wushaichthys*
00110110-0021-010???0?????????10100010011000120100000000-00101000010?000??1?012?????000010000020000??110100111110100101001000001001000-1421102

*Moradebrichthys*
00110110-0021-010?????????????10??00100110001?0??????000-00101000010?0?0?????1??????000010000010?????11?100111110100000001000000001000?0421102

*Peripeltopleurus*
0011(01)11(01)-0021-010???0?????????10100010011000120100000000-001010000101000??1?012?????00001000002000???11?100111110100101011000001001000-0421302

*Thoracopterus*
00110110-1121-010???0?????????10100010011???100100000000-00101000010?000??1?????????00001001000000??011?111111110101???011110001001000?1??1302

*Potanichthys*
00110110-0121-010???0?????1?0010100010011000120110000000-001010000001000??1??12?000?0000100100?000?0011?11111111010110101111001----000?1311302

*Gigantopterus*
00110110-??21-0?0???0?????????10?0001?01??00120?????0000-001010?00?0?000??11????????0000?00100?0?0??01??111111110?01?0??1111001----000?????3??

*Thoracopterus*_*magnificus*
00110110-01?1-010???0?????????10?00010011?00???110000000-00101000000?000??1???2?00??00001001000000?0011?111111110101?0?01111011-----00?0301302

*Thoracopterus*_*martinisi*
00111-21-01?1-010???0?????????10100010011?00???110?0?000-00101000000?000??1??12?00??0000100100???0?0011?111111110?01?0?01?11011-----00?030?302

*Venusichthys*
00010000000210000???0?????????100000100110110--100000000-001010110100011??1?102?????01001000004010???010100111110100000000000001000000-2121224

*Habroichthys*_*broughi*
10011-21-0?21-010???0?????????10????11010-??0--??????1-0-0111-0011-0010???1?102??0??0001-0000050?0???01?101111110100010011110111001000----12--

*Caturus*
100100000112110011100?????1?00101100101010111211001011-100111-100000000100111021101?1001-00010000000000?00011210-00000000000000000-100---102--

*Amia*
10010000011210001110010101100010110010100-110--1001011-100111-10000000010011102110111001-00010000000000100011210-10000002111000000-100----02--

*Lepisosteus*
100100000112100011110001111000111100100110111101001011-100---------000110011001100110001-00010301000001100011010-100000121000000001000---201--

*Semionotus*
100100000112100011110?????1?00111100100010111201001011-100111-00201000110011101100111001-00010101000001110011110-100000000000000001010---001--

*Kyphosichthys*_*grandei*
10010000011210011???0?????????1111001001101112010010?1-100111-00200000010?1?101?????1001-000101000???00110011110-100000000001000000010---101--

*Leptolepis*
100100000112100000001111011011100000100110111101010001-110111-01000000011111102101011001-00000000101100110011110-10000000000000000-200---?02--

*Elops*
1001000001121100001011?101111010000010010-111001010001-110111-01000000011111102101011001-00000000111100100011210-10000000011000000-200----02--

**5. References to Supplementary Information**

**Arratia G. 1999.** The monophyly of Teleostei and stem-group teleosts. Consensus and disagreements. In: Arratia G, Schultze H-P, eds. *Mesozoic fishes 2 – systematics and fossil record*. München: Verlag Dr. Friedrich Pfeil, 265–334.

**Arratia G. 2013.** Morphology, taxonomy, and phylogeny of Triassic pholidophorid fishes (Actinopterygii, Teleostei). *Society of Vertebrate Paleontology Memoir (Supplement to Journal of Vertebrate Paleontology)* **13:** 1–138.

**Brough J. 1931.** The Triassic fishes of the Karroo System and some general considerations on the bony fishes of the Triassic period. *Proceedings of the Zoological Society* **1931:** 235–296.

**Bürgin T. 1992.** Basal ray-finned fishes (Osteichthyes; Actinopterygii) from the Middle Triassic of Monte San Giorgio (Canton Tessin, Switzerland). *Schweizerische Palaontologische Abhandlungen* **114:** 1–164.

**Cartanyà J, Fortuny J, Bolet A, Mutter RJ. 2015.** *Colobodus giganteus* (Beltan, 1972) comb. nov. from the Upper Muschelkalk facies of Catalonia (NE Iberian Peninsula). *Neues Jahrbuch für Geologie und Paläontologie, Abhandlungen* **278:** 323–333 DOI 10.1127/njgpa/2015/0532.

**Cartanyà J, Fortuny J, Bolet A, and Garcia Artigas R. 2019.** Moradebrichthys vilasecae gen. et sp. nov., a new perleidid (Actinopterygii: Osteichthyes) from the Middle Triassic of Catalonia (NE Iberian Peninsula). *Neues Jahrbuch für Geologie und Paläontologie-Abhandlungen,* 2019, vol **292**: 171-190.

**Cavin L. 2010.** Diversity of Mesozoic semionotiform fishes and the origin of gars

(Lepisosteidae). *Naturwissenschaften* **97:** 1035–1040 DOI 10.1007/s00114-010-0722-7.

**Cloutier R, Arratia G. 2004.** Early diversification of actinopterygians; In: Arratia G, Wilson MVH, Cloutier R, eds. *Recent Advances in the Origin and Early Radiation of Vertebrates*. München: Verlag Dr. Friedrich Pfeil, 217–270.

**Coates MI. 1999.** Endocranial preservation of a Carboniferous actinopterygian from Lancashire, UK, and the interrelationships of primitive actinopterygians. *Philosophical Transactions of the Royal Society of London B* **354:** 435–462.

**Dai Y, Sun Z, Lu H, Jiang D, and Zhou M. 2021.** Redescription of *Chaohuperleidus* *Primus* (Actinopterygii, Perleidiformes) from Lower Triassic of Anhui Province, South China. *Beijing Da Xue Xue Bao* **57**:852-864.

**Forey PL. 1973.** A revision of the elopiform fishes, fossil and Recent. *Bulletin of the British Museum (Natural History)*, *Geology* **10(Suppl):** 1–222.

**Gardiner BG. 1984.** The relationships of the palaeoniscid fishes, a review based on new specimens of *Mimia* and *Moythomasia* from the Upper Devonian of Western Australia. *Bulletin of the British Museum (Natural History), Geology* **37:** 173-428.

**Gardiner BG, Maisey JG, Littlewood DTJ. 1996.** Interrelationships of basal neopterygians. In: Stiassney MLJ, Parenti LR, Johnson GD, eds. *Interrelationships of fishes*. San Diego: Academic Press, 117–146.

**Gardiner BG, Schaeffer B. 1989.** Interrelationships of lower actinopterygian fishes. *Zoological Journal of the Linnean Society* **97:**135–187 DOI 10.1111/j.1096-3642.1989.tb00550.x.

**Gardiner BG, Schaeffer B, Masserie JA. 2005.** A review of the lower actinopterygian phylogeny. *Zoological Journal of the Linnean Society* **144:** 511–525.

**Geng B-H, Jin F, Wu F-X, Wang Q. 2012.** New perleidid fishes from the Middle Triassic strata of Yunnan Province. *Geological Bulletin of China* **31:** 915–927.

**Grande L, Bemis WE. 1998.** A comprehensive phylogenetic study of amiid fishes (Amiidae) based on comparative skeletal anatomy: An empirical search for interconnected patterns of natural history. *Society of Vertebrate Paleontology Memoir (supplement to Journal of Vertebrate Paleontology)* **4:**1–690.

**Grande L. 2010.** An empirical synthetic pattern study of gars (Lepisosteiformes) and closely related species, based mostly on skeletal anatomy. The resurrection of Holostei. *Copeia* **10 (Supplement):** 1–871.

**Griffith J. 1977.** The Upper Triassic fishes from Polzberg bei Lunz, Austria. *Zoological Journal of the Linnean Society* **60:** 1–93 DOI 10.1111/j.1096-3642.1977.tb00834.x.

**Hilton EJ, Forey PL. 2009.** Redescription of †*Chondrosteus acipenseroides* Egerton, 1858 (Acipenseriformes, †Chondrosteidae) from the lower Lias of Lyme Regis (Dorset, England), with comments on the early evolution of sturgeons and paddlefishes. *Journal of Systematic Palaeontology* **7:** 427–453.

**Hilton EJ, Grande L, William EB. 2011.** Skeletal anatomy of the shortnose sturgeon, *Acipenser brevirostrum* Lesueur, 1818, and the systematics of sturgeons (Acipenseriformes, Acipenseridae). *Fieldiana–Life Earth Sciences* **3:** 1–168

**Hutchinson P. 1973a.** *Pseudobeaconia*, a perleidiform fish from the Triassic Santa Clara Formation, Argentina. *Breviora* **398:**1–24.

**Hutchinson P. 1973b.** A revision of the redfieldiiform and perleidiform fishes from the Triassic of Bekker’s Kraal (South Africa) and Brookvale (New South Wales). *Bulletin of the British Museum of Natural History (Geology)* **22:** 235–354.

**Lehman JP. 1952.** Etude complémentaire des possions de l’Eotrias de Madagascar. *Kungliga Svenska Vetenskapsakademiens Hangdlingar* **2:** 1–201.

**Lehman JP. 1979.** Note sur les Poissons du Trias de Lunz. I. *Thoracopterus* Bronn. *Annalen des Naturhistorischen Museums in Wien* **82:** 53–66

**Lin H-Q, Sun Z-Y, Tintori A, Lombardo C, Jiang D-Y, Hao W-C. 2011.** A new species of *Habroichthys* Brough, 1939 (Actinopterygii; Peltopleuriformes) from the Pelsonian (Anisian, Middle Triassic) of Yunnan Province, South China. *Neues Jahrbuch für Geologie und Paläontologie, Abhandlungen* **262:** 79–89 DOI 10.1127/0077-7749/2011/0186.

**Lombardo C. 2001.** Actinopterygians from the Middle Triassic of northern Italy and Canton Ticino (Switzerland): anatomical descriptions and nomenclatural problems. *Rivista Italiana di Paleontologia e Stratigrafia* **107:** 345–369.

**López-Arbarello A. 2012.** Phylogenetic interrelationships of ginglymodian fishes (Actinopterygii: Neopterygii). *PLoS ONE* **7:** e39370.

**López-Arbarello A, Zavattieri AM. 2008.** Systematic revision of *Pseudobeaconia* Bordas, 1944, and *Mendocinichthys* Whitley, 1953 (Actinopterygii: ‘Perleidiformes’) from the Triassic of Argentina. *Palaeontology* **51:** 1025–1052 DOI 10.1111/j.1475-4983.2008.00806.x.

**Ma X-Y, Xu G-H, Geng B-H. 2021.** *Feroxichthys panzhouensis* sp. nov., a hump-backed colobodontid (Neopterygii, Actinopterygii) from the early Middle Triassic of Panzhou, Guizhou, China. *PeerJ* **9:** e11257 DOI 10.7717/peerj.11257.

**Marramà G, Lombardo C, Tintori A, Carnevale G. 2017.** Redescription of ‘*Perleidus*’ (Osteichthyes, Actinopterygii) from the Early Triassic of northwestern Madagascar. *Rivista Italiana di Paleontologia e Stratigrafia* **123:** 219–242.

**Mutter RJ. 2002.** Revision of the Triassic family Colobodontidae sensu Andersson, 1916 (emended) whith a tentative assessment of perleidiform interrelationships (Actinopterygii: Perleidiformes). Unpublished Ph.D. thesis, Universität Zürich, Switzerland.

**Mutter RI. 2004.** The “perleidiform” family colobodontidae: A review. In Arratia G, Tintori A, eds. *Mesozoic Fishes 3 – Systematics, Paleoenvironments and Biodiversity.* München: Verlag Dr. Friedrich Pfeil, 197–208.

**Neuman AG, Mutter RJ. 2005.** *Helmolepis cyphognathus*, sp. nov., a new platysiagid actinopterygian from the lower Triassic Sulphur Mountain Formation (British Columbia, Canada). *Canadian Journal of Earth Sciences* **42:** 25–36.

**Nielsen E. 1942.** Studies on Triassic fishes from East Greenland. I. *Glaucolepis* and *Boreosomus*. *Meddeleser om Grønland* **138:** 1–403.

**Nielsen E. 1949.** Studies on Triassic fishes from East Greenland. II. *Australosomus* and *Birgeria*. *Meddeleser om Grønland* **146:** 1–309.

**Olsen PE, McCune AR. 1991.** Morphology of the *Semionotus elegans* species group from the Early Jurassic part of the Newark Supergroup of eastern North America, with comments on the family Semionotidae (Pisces: Neopterygii). *Journal of Vertebrate Paleontology* **11:** 269–292.

**Patterson C. 1975.** The braincase of pholidophorid and leptolepid fishes, with a review of the actinopterygian braincase. *Philosophical Transactions of the Royal Society of London*, *Series B* **269:** 275–579.

**Pinna MCC. 1996.** Teleostean monophyly. In: Stiassny MLJ, Parenti LR, Johnson GD eds. *Interrelationships of Fishes*. San Diego: Academic Press, 147–162.

**Schaeffer B, McDonald NG. 1978.** Redfieldiid fishes from the Triassic-Liassic Newark Supergroup of eastern North America. *Bulletin of the American Museum of Natural History* **159:** 129–173.

**Sun Z-Y, Lombardo C, Tintori A, Jiang D-Y, Hao W-C, Sun Y-L, Lin H-Q. 2012.** *Fuyuanperleidus* *dengi* Geng et al. 2012 (Osteichthyes, Actinopterygii) from the Middle Triassic of Yunnan Province, South China. *Rivista Italiana di Paleontologia e Stratigrafia* **118:** 359–373.

**Sun Z-Y, Tintori A, Jiang D-Y**, **Lombardo C**, **Rusconi M, Hao W-C, Sun Y-L. 2009.** A new perleidiform (Actinopterygii, Osteichthyes) from the Middle Anisian (Middle Triassic) of Yunnan, South China. *Acta Geologica Sinica* **83:** 460*–*470.

**Sun Z-Y, Tintori A, Lombardo C**, **Jiang D-Y**, **Hao W-C, Sun Y-L, Wu F-X, Rusconi M. 2008.** A new species of the genus *Colobodus* Agassiz, 1844 (Osteichthyes, Actinopterygii) from the Pelsonian (Anisian, Middle Triassic) of Guizhou, South China. *Rivista Italiana di Paleontologia e Stratigrafia* **114:** 363–376.

**Sun Z, Tintori A, Jiang D, and Motani R. 2013.** A new Perleididae from the Spathian (Olenekian, Early Triassic) of Chaohu, Anhui Province, China. *Rivista Italiana di Paleontologia e Stratigrafia* **119:**157-164.

**Tintori A.** **1990.** *Dipteronotus olgiatii* n. sp. (Actinopterygii, Perleidiformes) from the Kalkschieferzone of Ca’ del Frate (N. Italy). *Atti Ticinensi di Scienze della Terra* **33:** 191–197.

**Tintori A, Sassi D. 1992.** *Thoracopterus* Bronn (Osteichthyes: Actinopterygii): a gliding fish from the Upper Triassic of Europe. *Journal of Vertebrate Paleontology* **12:** 265–283 DOI 10.1080/02724634.1992.10011459.

**Tintori, A., Lombardo, C. 1996.** *Gabanellia* *agilis* gen. n. sp. n., (Actinopterygii, Perleidiformes) from the Calcare di Zorzino of Lombardy (North Italy). Rivista Italiana di Paleontologia e Stratigrafia, **102(2):** 227-236.

**Wade RT. 1935.** The Triassic Fishes of Brookvale, New South Wales. British Museum (Natural History), London, 110 pp.

**Wen W, Hu S**-**X, Zhang Q**-**Y, Benton MJ, Kriwet J, Chen Z-Q, Zhou C-Y, Xie T, Huang J-Y**. **2019.** A new species of *Platysiagum* from the Luoping Biota (Anisian, Middle Triassic, Yunnan, South China) reveals the relationship between Platysiagidae and Neopterygii. *Geological Magazine* **156:** 669–682 DOI 10.1017/S0016756818000079.

**Xu G-H. 2020a.** *Feroxichthys yunnanensis* gen. et sp. nov. (Colobodontidae, Neopterygii), a large durophagous predator from the Middle Triassic (Anisian) Luoping Biota, eastern Yunnan, China. *PeerJ* **8:** e10229 DOI 10.7717/peerj.10229.

**Xu G-H. 2020b.** A new species of *Luganoia* (Luganoiidae, Neopterygii) from the Middle Triassic Xingyi Biota, Guizhou, China. *Vertebrata PalAsiatica* **58:** 267–282 DOI 10.19615/j.cnki.1000-3118.200624.

**Xu G-H. 2021.** A new stem-neopterygian fish from the Middle Triassic (Anisian) of Yunnan, China, with a reassessment of the relationships of early neopterygian clades. *Zoological Journal of the Linnean Society* **191:** 375–394 DOI 10.1093/zoolinnean/zlaa053.

**Xu G-H, Gao K-Q. 2011.** A new scanilepiform from the Lower Triassic of northern Gansu Province, China, and phylogenetic relationships of non-teleostean Actinopterygii. *Zoological Journal of the Linnean Society* **161:** 595–612.

**Xu G-H, Gao K-Q, Coates MI. 2015a.** Taxonomic revision of *Plesiofuro mingshuica* from the Lower Triassic of northern Gansu, China, and the relationships of early neopterygian clades. *Journal of Vertebrate Paleontology* **35:** e1001515 DOI 10.1080/02724634.2014.1001515.

**Xu G-H, Gao K-Q, Finarelli JA. 2014.** A revision of the Middle Triassic scanilepiform fish *Fukangichthys longidorsalis*from Xinjiang, China, with comments on the phylogeny of the Actinopteri. *Journal of Vertebrate Paleontology* **34:** 747–759.

**Xu G-H, Ma X-Y. 2016.** A Middle Triassic stem-neopterygian fish from China sheds new light on the peltopleuriform phylogeny and internal fertilization. *Science Bulletin* **61:** 1766–1774.

**Xu G-H, Ma X-Y, Zhao L-J. 2018.** A large peltopleurid fish from the Middle Triassic (Ladinian) of Yunnan and Guizhou, China. *Vertebrata PalAsiatica* **56:** 106–120.

**Xu G-H, Wu F-X. 2012.** A deep-bodied ginglymodian fish from the Middle Triassic of eastern Yunnan Province, China, and the phylogeny of lower neopterygians. *Chinese Science Bulletin* **57:**111−118 DOI 10.1007/s11434-011-4719-1.

**Xu G-H, Zhao L-J. 2016.** A Middle Triassic stem-neopterygian fish from China shows remarkable secondary sexual characteristics. *Science Bulletin* **61:** 338–344 DOI 10.1007/s11434-016-1007-0.

**Xu G-H, Zhao L-J, Gao K-Q, Wu F-X. 2012.** A new stem-neopterygian fish from the Middle Triassic of China shows the earliest over-water gliding strategy of the vertebrates. *Proceedings of the Royal Society B* **280:** 20122261 DOI 10.1098/rspb.2012.2261.

**Xu G-H, Zhao L-J, Shen C-C. 2015b.** A Middle Triassic thoracopterid from China highlights the evolutionary origin of over-water gliding in early ray-finned fishes. *Biology Letters* **11:** 20140960 DOI 10.1098/rsbl.2014.0960.

**Zhao L-J, Lu L-W. 2007.** A new Genus of Early Triassic Perleidid Fish from Changxing, Zhejiang,

China. *Acta Palaeont Sin* **46(2)**:238-243.

**Zhou Z-H. 1992.** Review on *Peipiaosteus* based on new material of *P. pani.* *Vertebrata PalAsiatica* **30:** 85–101.
